# Balancing accuracy and interpretability of machine learning approaches for radiation treatment outcomes modeling

**DOI:** 10.1259/bjro.20190021

**Published:** 2019-07-04

**Authors:** Yi Luo, Huan-Hsin Tseng, Sunan Cui, Lise Wei, Randall K. Ten Haken, Issam El Naqa

**Affiliations:** Department of Radiation Oncology, University of Michigan, 519 W William Street, Ann Arbor, MI, USA

## Abstract

Radiation outcomes prediction (ROP) plays an important role in personalized prescription and adaptive radiotherapy. A clinical decision may not only depend on an accurate radiation outcomes’ prediction, but also needs to be made based on an informed understanding of the relationship among patients’ characteristics, radiation response and treatment plans. As more patients’ biophysical information become available, machine learning (ML) techniques will have a great potential for improving ROP. Creating explainable ML methods is an ultimate task for clinical practice but remains a challenging one. Towards complete explainability, the interpretability of ML approaches needs to be first explored. Hence, this review focuses on the application of ML techniques for clinical adoption in radiation oncology by balancing accuracy with interpretability of the predictive model of interest. An ML algorithm can be generally classified into an interpretable (IP) or non-interpretable (NIP) (“black box”) technique. While the former may provide a clearer explanation to aid clinical decision-making, its prediction performance is generally outperformed by the latter. Therefore, great efforts and resources have been dedicated towards balancing the accuracy and the interpretability of ML approaches in ROP, but more still needs to be done. In this review, current progress to increase the accuracy for IP ML approaches is introduced, and major trends to improve the interpretability and alleviate the “black box” stigma of ML in radiation outcomes modeling are summarized. Efforts to integrate IP and NIP ML approaches to produce predictive models with higher accuracy and interpretability for ROP are also discussed.

## introduction

Radiotherapy treatment in general and personalized adaptive radiotherapy (pART) in particular have a promising prospective to improve cancer patients’ therapeutic satisfaction.^1,2^ However, pART success highly depends upon accurate radiation outcomes prediction. Closely related to computational statistics, machine learning (ML) explores the study design and the construction of computer algorithms to learn from data and make data-driven predictions by employing complex mathematical optimization schemes.^3,4^ As more biophysical data become available before and during radiation treatment, the application of ML in radiation oncology will continue to experience tremendous growth, including treatment planning optimization,^5,6^ normal tissue toxicity prediction,^7,8^ tumor-response modeling,^9,10^ radiation physics quality assurance.^11–13^ In this paper, we focus on the application of ML approaches in radiation outcome modeling as a case study.

ML is typically classified into supervised learning, unsupervised learning and reinforcement learning methods.^14^ While supervised learning handles a set of data containing both the inputs and the labeled outputs, unsupervised learning deals with a set of data with only inputs. Reinforcement learning intends to identify the best actions in a dynamic system by maximizing the cumulative reward. As multilayer neural networks to extract “complex patterns” from large scale of data sets become popular in the era of Big Data, ML approaches can also be categorized into shallow learning and deep learning (DL), where the latter combines data representation with classification/regression tasks in the same framework. As DL has garnered remarkable attention for its capacity to achieve accurate prediction in various fields, there is a growing realization that better explanation of these ML methods is equally desired. While explainability and interpretability have been used interchangeably, we would like to distinguish between them to provide more accurate definition of different ML techniques in this review. Explainable models can be defined as those that are able to summarize the reasons for the behavior of ML algorithms, gain the trust of users, or allow the user to produce insights into the causes of the algorithm decisions.^15^ Essentially, one consensus among recent studies is that explainability based on human understanding is not a monolithic concept, but rather a complex construction. According to the description of Gilpin et al and Ribeiro et al,^15,16^ it can be decomposed into several human factors, such as trust, causality, transferability, and algorithm transparency. On the other hand, interpretability can be loosely defined as comprehending what a model did (or might have done) based on the inputs, with the capacity to defend its actions, provide relevant responses to questions, and be audited. Their relationship can be described as that explainable models are interpretable by default and the reverse is not always true. Although interpretability alone is insufficient for the explanation of different ML techniques, it is a necessary first step towards full explainability, and it is employed in this paper to classify existing literature in radiation oncology. In this context, ML approaches can be sorted into interpretable (IP) and non-interpretable (NIP) approaches. In addition to DL, some shallow learning approaches such as support vector machines (SVMs), random forests (RFs), and “shallow” neural network approaches belong to the NIP category. The rest of shallow learning approaches such as generalized linear models (*e.g.* linear regression, logistic regression), linear discriminant analysis, decision trees, Bayesian networks are considered IP ML approaches.

For clinical applications such as radiation outcomes prediction, the accuracy and interpretability of the ML approaches are major concerns. As accurate prediction of the treatment outcomes provides direct guidance to tailor and adapt a treatment plan in cancer therapy, and it is highly essential to use interpretable results for clinical decision-making support. If the goal is to assist physicians and patients reach the best decision, then an ML approach with a good balance between interpretability of the results and accurate predictions is needed to gain trust of the treating clinician, *i.e.* increase its credibility.^17,18^ However, no single IP or NIP approach is located at a Pareto optimum, where it enjoys both the highest accuracy and the highest interpretability, but it rather exists as a comprise between them. For example, while a decision tree has more interpretable capability than the RF approach, its accuracy is generally outperformed by the latter.

The relationship of IP and NIP ML approaches in terms of accuracy and interpretability has been studied.^19^ However, the selected ML approaches refer to “off the shelf” algorithms, where they have been implemented by someone else and are available in prepackaged libraries. In other words, there will be some room to improve their accuracy or interpretability performance. In fact, researchers in the field of medical physics have been struggling to improve accuracy and interpretability of the ML approaches for radiation outcomes prediction, and their efforts were based upon both the IP and NIP ML categories. This study intends to summarize current efforts and to provide a big picture of the current trends to develop more advanced ML approach for pART. The rest of the paper is organized as follows. Section 2 introduces the strategies that have been used to increase the accuracy of IP ML approaches, Section 3 summarizes the developed strategies or tactics to improve interpretability of NIP ML approaches, discussion and conclusions are given in Section 4.

## balancing accuracy and interpretability of *interpretable* ML approaches

### Logistic regression

Logistic regression is most commonly used model to represent linear relationships with the assumption of uncorrelated features. For example, logistic regression has been employed to predict xerostomia after radiotherapy, and in some instances it can approximate the performance of neural networks when sigmoidal functions are used.^20^ An objective statistical multivariate model was also developed to describe radiation pneumonitis risk by assessing continuous and nominal parameters to determine the optimal model order and its parameters.^21^ In order to predict pneumonia risk and hospital 30-day readmission, generalized additive models (GAMs) with low-dimensional terms were developed, and pairwise interactions were added to standard GAMs resulting in GA^2^Ms. Logistic regression with single and cross-terms not only improved accuracy compared to the GAMs, the pairwise interaction could also be visualized as a heat map^22^. It turns out adding pairwise cross-terms may improve the prediction accuracy of the logistic regression, although it might not be fully explainable.

For modern data sets with a high dimension of features, GAMs and GA^2^Ms could be very complicated by considering all the features and their interactions. While ridge regression (logistic regression +L2 norm regularization) intends to regularize the ill-posed problems caused by high dimensional data sets,^22^ least absolute shrinkage and selection operator (LASSO, logistic regression + L1 norm regularization), is a regression analysis method that performs both variable selection and regularization to enhance the prediction accuracy and interpretability of the statistical model it produces.^23^ Elastic net is another regularized regression model that linearly combines the L1 and L2 penalties of the LASSO and ridge methods to handle correlated features and high-dimensional data set, and it was used for outcome prediction in chemoradiotherapy.^24^ The elastic net was reported to have similar prediction performance as RFs and yielded higher discriminative performance than decision tree, neural network, SVM and LogitBoost in chemoradiotherapy outcome and toxicity prediction, particularly, when the complexity of the input features is limited to basic clinical and dosimetric variables.^24^ However, with the increment of the accuracy, elastic net trades off a little interpretability compared to logistic regression.^9^

In order to facilitate the interpretability of regression-based analyses, graphical calculating devices named “nomograms” were widely employed in clinical practice for oncology applications including radiation treatment outcomes prediction by conducting the approximate graphical computation of a regression function.^25^ The group at Memorial Sloan Kettering Cancer Center has developed several nomograms for varying cancer diagnostics.^26^ In addition, such nomograms have been used to predict response for treatment. For instance, a nomogram was devised for estimating treatment failure among males with clinically localized prostate cancer treated with radical prostatectomy^27^ and for predicting disease-specific survival after hepatic resection for metastatic colorectal cancer.^28^ Nomograms were also employed to predict recurrence-free survival for cervical cancer based on combining individual clinical information with imaging-based fludeoxyglucose/positron emission tomography prognostic factors.^29^

### Decision tree

Decision trees can model nonlinear effects and are obviously interpretable as long as the tree depth is shallow.^24^ More than three decades ago, a recursive algorithm (decision tree) was applied to arbitrary dose–volume histograms to estimate the complication probability for treatment planning optimization.^30^ Recently, a recursive partitioning analysis was constructed to stratify patients into risk groups for clinically significant radiation pneumonitis after chemoradiation therapy for lung cancer.^31^ Additionally, decision trees were employed to predict pneumonitis in Stage I non-small cell lung cancer (NSCLC) patients after stereotactic body radiation therapy (SBRT). Ensemble techniques based on the decision tree such as boosting with RUSBoost and bagging with RFs had been used to improve its accuracy, but at the expense of losing its interpretability.^7^

In order to study weight loss in head and neck cancer patients treated with radiation therapy, a classification and regression tree (CART) prediction model was developed based on a knowledge-discovery approach. The CART not only does not require a specification of the function to model covariates, but also its prediction accuracy increases with additional treatment toxicity information.^32^ It seems that tree structure has a good potential to interpret nonlinear relationships and to be integrated with other NIP ML approaches for prediction accuracy improvement. Gradient boosting machine (GBM) intends to produces a prediction model by combing weak prediction decision trees, and it has been employed to predict long-term meningioma,^9^ outcomes after radiosurgery for cerebral arteriovenous malformations with a high prediction performance and a less interpretability.^33^ As a tree-structured boosting, MediBoost is a new framework to construct decision trees that retain interpretability while having accuracy similar to ensemble methods.^34^ While it has the same structure as CART to build a single decision tree, it has the improved accuracy by considering weighted versions of all cases at each split.^9^

### Bayesian network

Naïve Bayesian network (NBN) is a simple probabilistic classifier based on applying Bayes’ theorem with strong (naive) independence assumptions, and it is interpretable but less accurate.^35^ An advantage of the NBN is that it requires a small amount of training data to estimate the parameters (means and variances of the variables) necessary for classification. Since independent variables are assumed, only the variances of the variables for each class need to be determined instead of the entire covariance matrix.^36^ Hierarchical Bayesian networks (HBNs) are an extension of NBNs, which intends to improve inference and learning methods by using knowledge about the structure of the data. In order to predict 2-year survival in lung cancer patients treated with radiotherapy, HBN models were developed, and they were reported to outperform SVM models at handling missing data, and therefore are more suitable for the medical domain.^37^ In a study of modeling local failure in lung cancer, a graphical HBN framework was generated to demonstrate that combining physical and biological factors with a suitable framework can improve the overall prediction, which highlights the potential of the integrated approach to predict post-radiotherapy local failure in NSCLC patients.^38^ Additionally, a HBN was employed to estimate overall survival among colon cancer patients in a large population-based data set, resulting in a significant improvement upon existing AJCC stage-specific OS estimates.^39^

**Figure 1.**
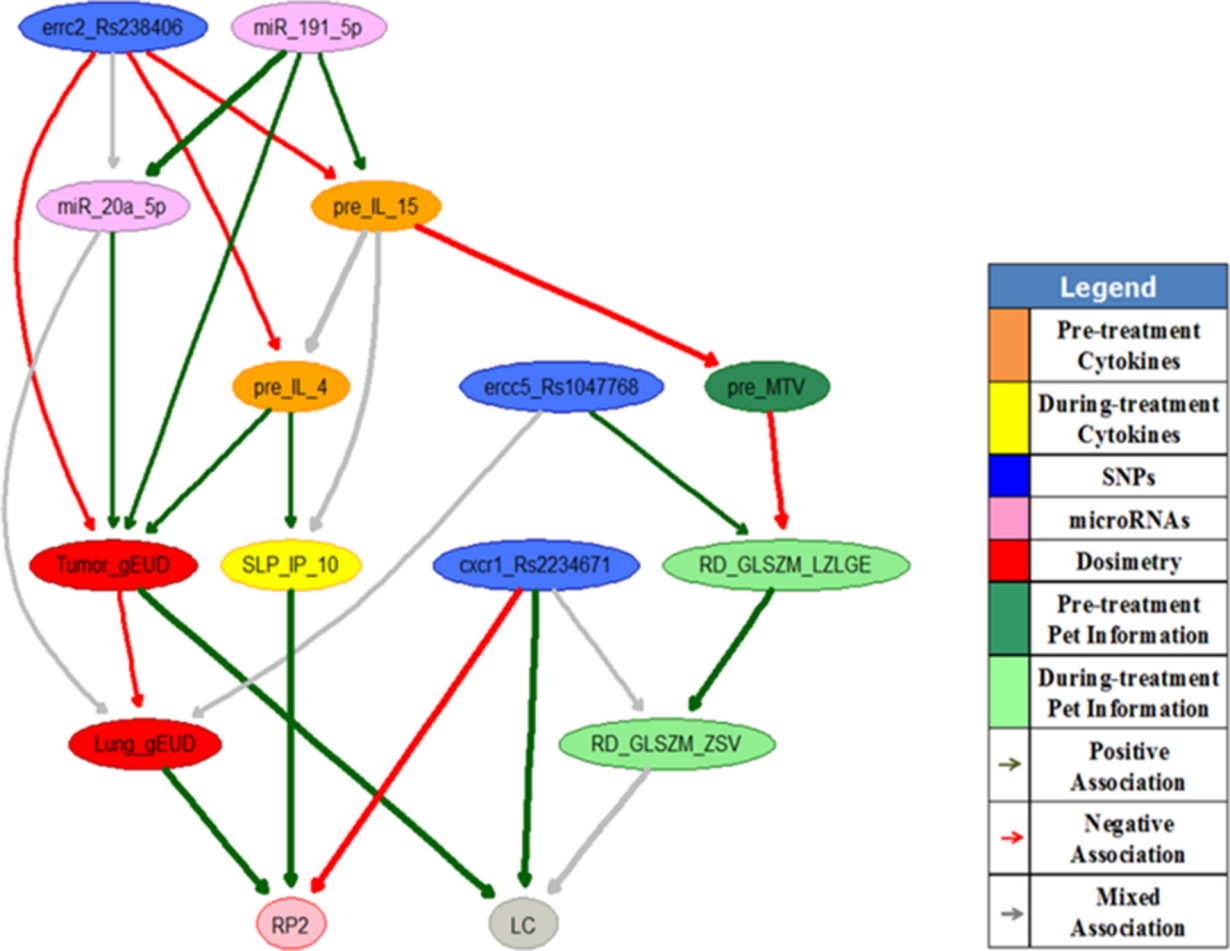
A during treatment MO-HBN for LC and RP2 prediction in lung cancer.^40^ RP2, radiation pneumonitis toxicity Grade II or above; LC, local control; MO-HBN, multiobjective hierarchical Bayesian network.

Moreover, a multiobjective HBN (MO-HBN) was developed to explore the biophysical relationships among treatment plans, patients’ personal characteristics and radiation outcomes so that appropriate treatment plans before and during the course of radiotherapy can be identified.^40^ Figure 1 shows an example of a during treatment MO-HBN to predict tumor local control (LC) and radiation pneumonitis toxicity Grade II or above (RP2) simultaneously in lung cancer patients. The important features for radiation outcomes prediction including tumor and lung gEUDs, three SNPs (errc2_Rs238406, ercc5_Rs1047768 and cxcr1_Rs2234671), two miRNAs (miR_20a_5p and miR_191_5p), two pre-treatment cytokines (IL_15 and IL_4), one pre-treatment radiomics feature (MTV), the relative change of one during treatment cytokine (IP_10) and the relative changes of two during treatment radiomics features (GLSZM_LZLGE, GLSZM_ZSV) were selected from a retrospective data set as denoted by the nodes in the figure. The edges of the MO-HBN, denoted by different colors, represent the biophysical relationships between the features analyzed. The study demonstrated that the MO-HBN has the potential to achieve a better performance than that of the corresponding NBN due to its hierarchical structure and additional biophysical information, and its prediction performance can be improved with patients’ response during radiotherapy.^40^ However, the confidence interval of the MO-HBN’s prediction performance is still relatively large.

Although the BNs do not offer a significant improvement in outcome prediction over those resulting from less complex classifier algorithm as naïve Bayes, logistic regression or C4.5 decision trees, they still provide unique benefits to explore the relationship among features from large patient cohort data. The ability of carrying out causal inferences allows them to be utilized for answering complex clinical questions from unobserved evidence, and the probability distributions underlying the BN can be automatically updated with newly added patient information. Although it is hard to automatically learn a single graph that faithfully represents the casual structure of an application field, hybrid causal learning is an emerging field to show promise in obtaining causal structures with high prediction performance and causal patterns set out by domain experts (HBN with expert knowledge, HBN-EK).^41^

## balancing accuracy and interpretability of *non-interpretable* ML approaches

### Random forests and support vector machine

As previously stated, some shallow learning methods such as RFs and SVMs belong to the NIP ML approaches. RFs are an ensemble learning method which constructs a multitude of decision trees at training time and outputs the mean prediction of the individual trees. While variance can be controlled from the ensemble learning, the ensemble learning approach can sacrifice most of its interpretability at the same time, except that the frequency of feature appearance in the top layers of the ensemble decision tree may be used to explain their importance. However, the concept of RFs was integrated with other IP ML approaches to balance their accuracy and interpretability for radiation outcomes prediction. For example, formerly mentioned Mediboost approach^34^ attempts to emulate the performance of RFs while maintaining the intuition of classical decision trees.^42^

A SVM with a radial basis function kernel (SVM-RBF) transforms the original feature space into another space that can separate classes better. This transformation, however, can be much less intuitive than linear SVMs.^24^ A non-linear SVM was developed for prediction of local tumor control after Stereotactic Body Radiation Therapy for early-stage NSCLC, and the prediction performance of the SVM model was significantly larger than that of a logistic tumor control probability model.^43^ Interpreting SVM models is far from obvious, and the absence of a direct probabilistic interpretation also makes SVM inference difficult. However, work was done in providing methods to visualize SVM results as nomograms to support interpretability.^42^ A nonlinear kernel, called localized radial basis function kernel (SVM-LRBF) was developed with the assumptions of intrafeature nonlinearity and interfeature independence. In addition to capturing nonlinearity of the classification function, the LRBF kernel can be visualized via nomograms. The SVM-LRBF method together with other SVM methods with linear kernel and RBF kernel had been applied for breast cancer prediction, and the study showed that while all the three kernel methods were equal in performance in terms of the area under the curve in the ROC curve, LRBF kernel was less sensitive to noise features than an RBF kernel.^44^

### Deep learning

#### The impact of deep learning on radiation outcomes prediction

As a NIP ML approach, DL is mostly an extension of previously existing forms of artificial neural networks (ANNs) to larger number of hidden layers and artificial neurons in each layer for automatic discovery of useful features. Historically, ANNs lost popularity in favor of SVMs, RFs and gradient boosting trees due to the limited data set, computing resources and being prone to local minima. With the availability of larger data sets, graphical processing units (GPU) and stochastic gradient descent algorithms, DL became possible to explore faster the training of larger, deeper architectures.^45^ The key property of DL is that it can automatically learn useful representations of the data without conducting feature selection, which is one important component of other ML techniques. The reason why DL has relatively high prediction performance is that an architecture with sufficient depth can produce a compact representation, whereas an insufficiently deep one may require an exponentially larger architecture to represent the same function.

Now, DL based on neural networks has broad applications in radiation response, and it is poised to dominate medical image analysis for radiation outcomes prediction. Convolution neural networks (CNNs) were used to extract features from non-medical images for computer-aided diagnosis tasks,^46^ extract radiomics features from the image patterns in developing a radiomics-based predictive model,^47^ and extract deep features from preoperative multimodality MR images for survival prediction in Glioblastoma Multiforme.^48^ Also, a CNN was developed to analyze rectum dose distributions and to predict rectal toxicity. ^28^ Studies show that DL has better performance than shallow learning approaches in radiation outcomes prediction. As a methodological proof-of-principle, a deep neural network (DNN) was created to predict the complete response of advanced rectal cancer after neo-adjuvant chemoradiation, which is an accurate surrogate for long-term local control. The DNN outperformed a linear regression model and a SVM model.^49^ Also, CNNs and their variants were applied to the discovery of consistent patterns in three-dimensional dose plans associated with toxicities after liver stereotactic body radiotherapy. When the number of false negatives, *i.e.* missed toxicities, was minimized, DL produced almost two times fewer false‐positive toxicity predictions in comparison to dose–volume histogram‐based predictions.^50^

While the development of DL will transform the way we use imaging for diagnosis, treatment planning and decision making and will disrupt the way we practice medicine in a positive way, it may also affect clinical practice in negative ways.^49^ Given that the field of medical physics has unique characteristics that differentiate it from those fields where these techniques have been applied successfully, the DL techniques have their limitations and nuances in radiation oncology with the limited sample sizes.^51^ Another important issue with the DL is that these black-box-like networks are very difficult to debug, isolate the reason behind certain outcomes, and predict when and where failures will happen,^52^ which are called interpretability challenges.^53^ As the limited sample size issue could be gradually released by further collaboration of the hospitals and data centers, solving the issue of DL’s interpretability becomes more important. Actually, the underlying mathematical principles of DLs are understandable. But they lack an explicit declarative knowledge representation, hence have difficulty in generating the underlying explanatory structures.^54^ Although the potential of DL to improve prediction accuracy may outweigh their interpretability challenges in many industries,^32,54^ professionals in the field of radiation oncology are working mostly with distributed heterogeneous and complex sources of data, and there must be a possibility to make the results re-traceable on demand.^54^ There has been increasing interest in radiation oncology to make DL transparent, interpretable, and explainable, and the efforts to improve its interpretability for radiation outcomes prediction are summarized in the next section.

## interpretability improvement for deep learning

### Deep learning with a combination of handcrafted features and latent variables (DL-HLV)

**Figure 2.**
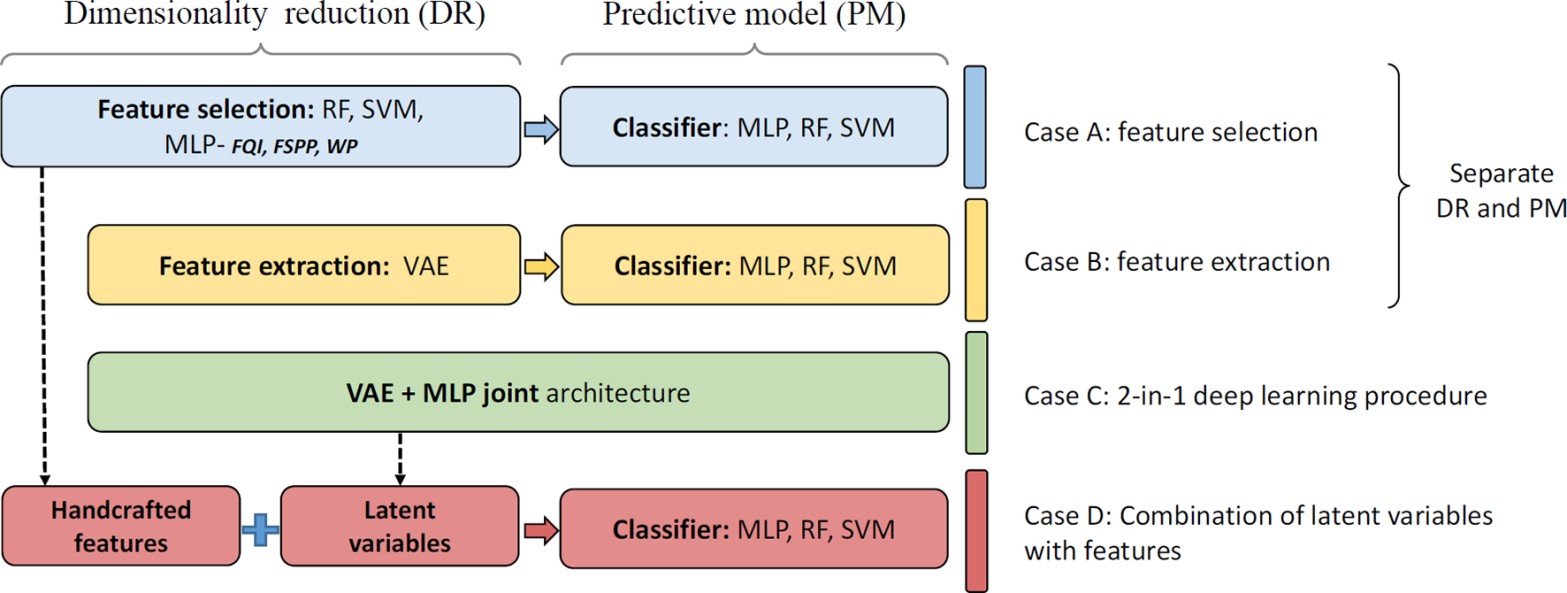
The evaluation of combination of handcraft features and latent variables for radiation-induced lung damage prediction,^56^ where, “RF”, random forest; “SVM”, support vector machine; “MLP”, multi-layer perceptron; “FQI”, feature quality index; “FSPP”, feature-based sensitivity of posterior probability; “WP”, weight pruning; “VAE”, variational autoencoders.

When a DNN is used as a feature extractor thousands of features are extracted. Unlike engineered handcrafted features, these features do not directly relate to something radiologists can easily interpret. Supplementing DL with information already known to be useful may improve the performance of these DL models and their interpretability. Previously, for survival prediction following glioblastoma multiforme, after deep features were extracted from preoperative multimodality MR images, a six-deep-feature signature was constructed by using the LASSO Cox regression model. The deep feature signature was combined with clinical risk factors to build a radiomics nomogram. The combined model not only achieved better performance for OS prediction, but also increased the interpretability of survival prediction through a nomogram construction.^48^ Similarly, a methodology was developed to extract and pool low- to middle-level features using a pretrained CNN and to fuse them with handcrafted radiomic features computed using conventional CADx methods. In comparison to existing methods, the fusion-based breast CADx method demonstrated statistically significant improvements in terms of area under the curve on three different imaging modalities, and can also be used to more effectively characterize breast lesions.^55^ Recently, the combination of traditional ML methods and DL variational autoencoders (VAE) techniques was developed to deal with limited datasets for radiation-induced lung damage prediction as shown in Figure 2.^56^ It was demonstrated that a multilayer perceptron (MLP) method using weight pruning (WP) feature selection achieved the best performance among different hand-crafted feature selection methods, and the combination of handcrafted features and latent representation (Case D: latent *Z* + WP + MLP) yielded significant prediction performance improvement compared with handcrafted features only (Case A: WP + MLP), VAE-MLP disjoint (Case B) and VAE-MLP joint architectures (Case C).

### Deep learning with sensitivity analysis (DL-SA)

**Figure 3.**
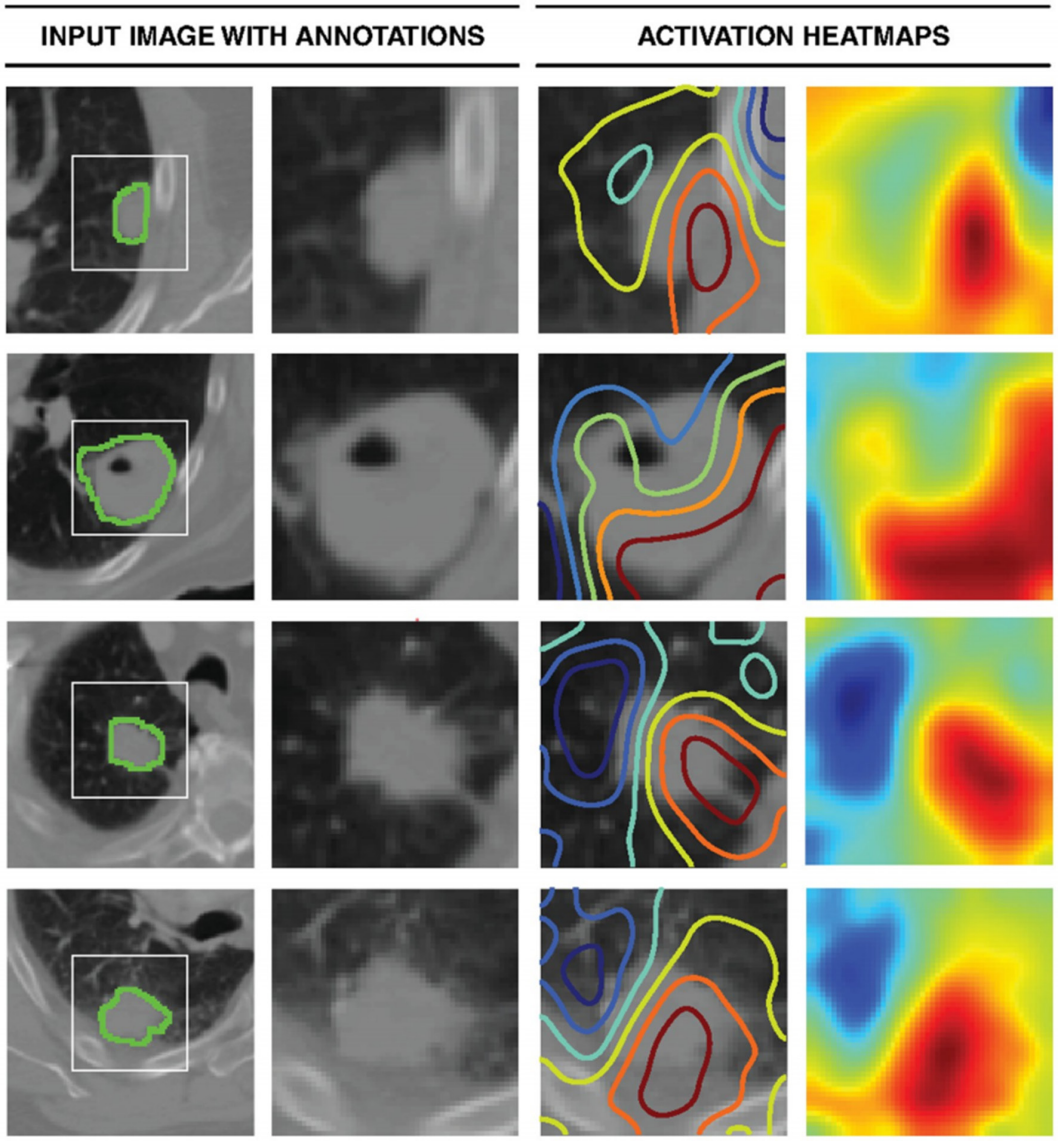
Activation mapping. The first column represents the central axial slice of the network input (150 × 150 mm) with tumor annotations. In the second column, a 50 × 50 mm patch is cropped around the tumor. In the third column, activation contours are overlaid, with blue and red showing the lowest and highest contributions (gradients), respectively. Column four represents the activation heatmaps for a better visual reference.^52^

Another method to increase the interpretability of DL is to calculate the sensitivity of the prediction with respect to changes in the input. Heat maps are visualization techniques that represent the importance of each pixel for the prediction task, which could help further optimize a CNN training approach. In a study of developing survival CNNs to predict cancer outcomes from histology and genomics, a heat map was employed to investigate the visual pattern that SCNN methods associate with poor outcomes by displaying the risks predicted by the SCNN in different regions of whole-slide images. The transparent heat map overlays in the study enable pathologists to correlate the predictions of highly accurate survival models with the underlying histology over the expanse of a whole-slide image. ^57^ In a DL-based radiomics model for survival prediction in glioblastoma multiforme, a heat map was also used to show the Z-score difference of each radiomics feature between high risk and low risk group, and a consistency of radiomics feature Z-Score between the discovery data set and the validation data set.^48^ In a retrospective multicohort radiomics study for lung cancer prognostication, DL was used in medical image for automated quantification of radiographic characteristics to improve patient stratification. A *gradient-weighted activation mapping* technique was employed to generate activation maps by mapping important regions in an input image with respect to predictions made, and the heat maps indicate regions in the input image having the most impact on the final prediction layer as shown in Figure 3.^52^ In a previous study to develop CNNs for individualized hepatobiliary toxicity prediction after liver stereotactic body proton therapy, *saliency maps* of the CNNs were used to estimate the toxicity risks associated with irradiation of anatomical regions of specific organs at risk, and the CNN saliency maps automatically estimated the toxicity risks for portal vein regions.^50^ In order to classify lung cancer using chest X-ray images, a 121-layer CNN was developed along with the transfer learning scheme. A *class-activation map* technique was employed to provide a heat map to identify the location of the lung nodule.^58^

### Deep learning with attention mechanisms (DL-AM)

Attention mechanisms are optional components of sequential prediction systems that allow the system to sequentially focus on different subsets of the input, and the subset selection is typically conditioned on the state of the system which is itself a function of the previously attended subsets. In addition to reducing the computational burden of processing high dimensional inputs by selecting only process subsets of the input, attention mechanisms also allow the system to focus on distinct aspects of the input and thus improve the ability to extract the most relevant information. Especially, soft attention mechanisms avoid a hard selection of which subsets of the input to attend and use a soft weighting of the different subsets for each piece of the output, thus leading to improvements in the quality of the generated outputs. The advantage brought by the soft-weighting is that it is readily amenable to efficient learning via gradient backpropagation.^59^ Additionally, a gated recurrent unit (GRU)-based recurrent neural network (RNN) with *hierarchical attention* (GRNN-HA) was developed for clinical outcomes prediction through handling the high dimensionality of medical codes, modeling the temporal dependencies of healthcare events and characterizing the hierarchical structure of healthcare data. It was reported to have a better prediction accuracy and improve the interpretability of predictive models compared to baseline models by using the diagnostic codes from the medical Information Mart for Intensive Care to evaluate the model. The interpretability of the model depends on attention weights assigned to individual diagnostic codes and hospital visits, which were determined from relative importance of diagnostic codes on prediction.^60^

### Deep learning with disentangled hidden layer representations (DL-DHLR)

**Figure 4.**
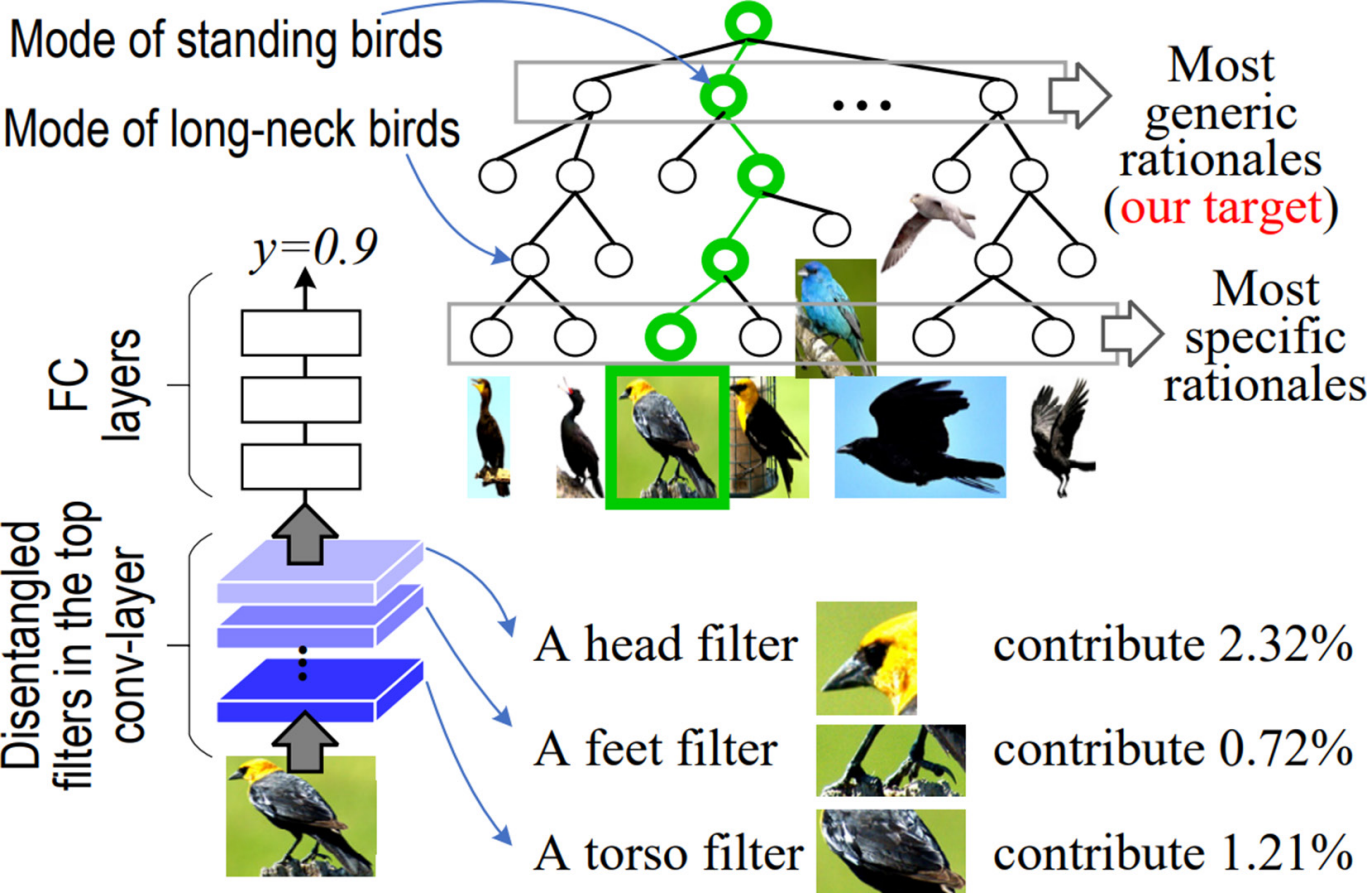
Decision tree that encodes all potential decision modes of the CNN in a coarse-to-fine manner. a CNN was learned for object classification with disentangled representations in the top convolution layer, where each filter represents an object part. For an input image, a parse tree (green lines) can be referred from the decision tree to semantically and quantitatively explain which object parts (or filters) are used for the prediction and how much an object part (or filter) contributes to the prediction.^63^CNN, convolution neural network.

Training DNNs with disentangled hidden layer representations is an active area of research to improve the interpretability of DL, although they have not been used for radiation outcomes prediction. The disentanglement of “the mixture of patterns” encoded in each filter of CNNs mainly disentangle complex representation in convolution-layers and transform network representations into interpretable graphs.^61^ An explanatory graph represents the knowledge hierarchy hidden in convolution-layers of a CNN. While each filter in a pre-trained CNN may be activated by different object parts, part patterns can be disentangled from each filter in an unsupervised manner to clarify the knowledge representation.^62^ A CNN was learned for object classification with disentangled representations in the top convolution-layer, where each filter represents a specific object part. Since the decision tree encodes various decision modes hidden inside fully connected layers of the CNN in a coarse-to-fine manner, given an input image, the decision tree infers a parse tree to quantitatively analyze rationales for the CNN prediction as shown in Figure 4.^63^ The above methods focus on the understanding of a pre-trained network, but it is more challenging to learn networks with disentangled representations.^64–66^

## discussion and conclusions

Interpretability and explainability are different concepts although they have been used interchangeably. While the former is about being able to discern the predictions without necessarily knowing the underlying mechanics, the latter is being able to quite literally explain what are the mechanics that led to a particular behavior or decision by the algorithm.^67^ In medicine including radiation oncology, interpretability represents physician’s ability to accept, and interpret an algorithm decision in a scientifically sound manner without the need to explain algorithmic behaviors. As questions of accountability and transparency become more and more important, the interpretability of AI algorithms for radiotherapy outcomes prediction has improved in recent years, but is still far away from achieving full explainability. In this study, we focus on the trade-off of accuracy and interpretability in evaluating the prediction performance of common ML approaches, and summarize the balance of IP and NIP ML approaches for radiation outcomes prediction purposes from the current radiation oncology literature.

Table 1 shows the accuracy, interpretability and explainability levels of basic ML approaches such as logistic regression, decision trees, naïve Bayesian networks, SVM kernels, DL, and improved ML approaches associated with them. Since understanding the reasons behind prediction is quite important in assessing trust or credibility if one plans to take a clinical action based on a prediction, an algorithm, called local interpretable model-agnostic explanations (LIME), was developed to interpret the predictions of any classifier by approximating it locally with an interpretable model.^69^ Due to its potential to enhance the interpretability of DL approaches, combining DL with LIME (DL-LIME) is considered as an improved DL approach as listed in Table 1. The number of “stars” associated to each ML approach in the table intends to describe the relative assessment of the accuracy, interpretability and explainability among these ML approaches, where the more stars represent the higher accuracy or interpretability or explainability. The evaluation of each ML approach is generated based on indicated literature next to it in the table. As can be seen from Table 1, the explainability of ML NIP methods is still a work in progress in many instances. It is interesting to evaluate the properties of these ML approaches in a general way by reviewing more literatures in different theoretic and application fields. However, it is out of the scope of this paper.

**Table 1.** The evaluation of the accuracy (A), interpretability (I) and explainability (E) of ML approaches in radiation outcomes prediction

**Basic ML**	**Type**	**A**	**I**	**E**	**Improved ML**	**Type**	**A**	**I**	**E**
*Logistic regression^20,21^*	IP	*	****	***	GA^2^M^68^	IP	**	***	**
					Ridge Regression^22^	IP	**	**	*
					LASSO^23^	IP	**	***	**
					Elastic Net^9,24^	IP	***	**	*
*Decision tree* ^24,30,31^	IP	**	*****	*****	CART^32^	IP	***	****	*****
					Random Forests^7^	NIP	****	*	NA
					GBM^9,33^	NIP	****	*	NA
					MediBoost^9,34^	IP	****	**	*
*Naïve BN* ^35,37^	IP	*	****	****	HBN^38,40^	IP	**	***	**
					HBN-EK^41^	IP	**	****	***
*Linear SVM* ^24^	NIP	**	**	*	SVM-RBF^43^	NIP	***	*	NA
					SVM-LRBF^44^	NIP	***	**	*
*Deep learning^49,50^*	NIP	****	*	NA	DL-HLV ^48,55,56^	NIP	*****	**	NA
					DL-SA^52,57^ /AM^59,60^	NIP	*****	**	NA
					DL-DHLR^61–63^	NIP	*****	***	NA
					DL-LIME^69^	NIP	*****	***	NA

BN, Bayesian network; CART, classification and regression tree; DHLR, disentangled hidden layer representation; DL-AM, deep learning withattention mechanisms; DL-HLV, deep learning withcombination of handcrafted features and latent variables; GBM, gradient boosting machine; HBN, hierarchical Bayesian network; HBN-EK, hierarchical Bayesiannetwork with expert knowledge; HLV, handcrafted features and latent variables; IP, interpretable; LASSO, least absolute shrinkage and selection operator; LIME, local interpretable model-agnostic explanation; ML, machine learning; NIP, non-interpretable; SVM, support vector machine.

**Figure 5.**
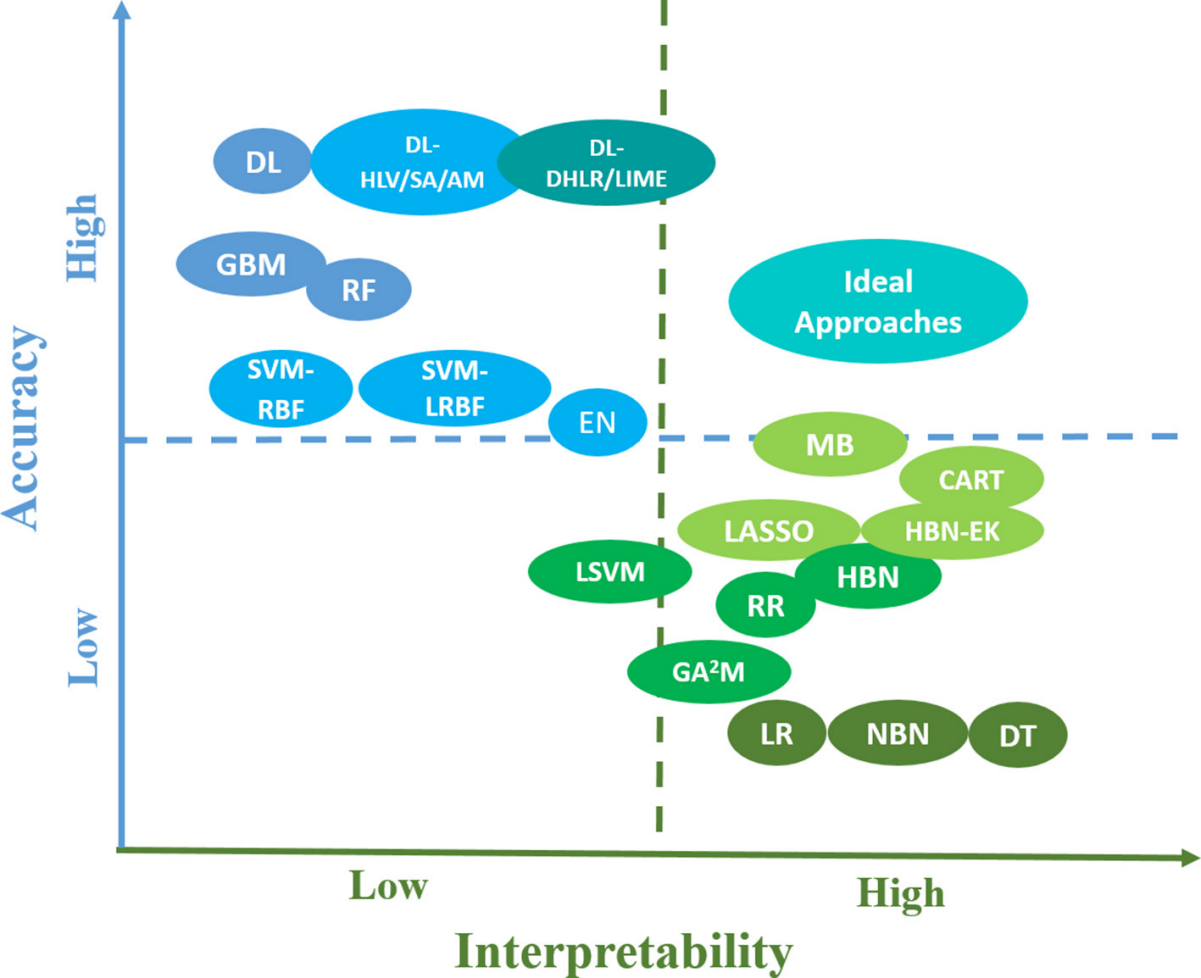
The accuracy and interpretability of IP and NIP ML approaches in radiation outcomes prediction and the location of potential ideal approaches with more balanced accuracy and interpretability for the pART. Besides the notifications introduced in the paper, the rest of abbreviations in the figure can be described as follows, “EN”, elastic net; “LR”, logistic regression; “MB”, MediBoost; “RR”, ridge regression; “LSVM”, linear support vector machine; “DT”, decision tree. IP, interpretable; ML, machine learning; NIP, non-interpretable.

Due to the limited data sizes in radiation treatment and the requirement of clinical decision-making for pART, developing unique ML approaches to achieve the Pareto optimum of accuracy and explainability is necessary and challenging at the same time. Explainable AI was proposed based on the trade-off between prediction accuracy and explainability by producing more explainable models and maintaining a high level of learning performance.^70^ However, as previously stated, we only focus on accuracy and interpretability, an initial stage towards full explainability, in this paper. The efforts to balance them not only came from IP ML approaches but also from NIP ML aspects as illustrated in Figure 5. The *y*- and *x*-axes of the figure represent the accuracy and interpretability of IP and NIP ML approaches for radiation outcomes prediction, respectively. Then the locations of these ML approaches were determined based on radiation oncology literatures as shown in Table 1. For the sake of clear description, blue or green color was used to represent NIP or IP ML approaches in terms of accuracy or interpretability. While the deeper the color indicates the higher accuracy or interpretability, ideal approaches to balance them are denoted as cyan color, which is the mixed color of blue and green. In addition to giving a general concept of the current status of ML approaches for radiation outcomes prediction, the figure also shows potentially possible trends to develop more balanced ML approach for pART.

The rising of DL approaches is attributed to their potential for high accuracy performance when sufficient data and computational support are available. These black box models create nonlinear predictors and automatically take into consideration a large number of implicit variable interactions. However, what makes them accurate is what makes their predictions difficult to understand; they are too complex. The exact DL architecture does not seem to be the most important determinant in getting a good solution for both accuracy and interpretability. A key aspect that is often overlooked is that expert knowledge about the task to be solved can provide advantages that can go beyond adding more layers to a CNN.^59^

On the other hand, the main purpose of a predictive model’s interpretability is to conduct statistical inferences, which intends to use the model to learn about the data generation process. However, none of NIP ML methods are able to conduct inference. In contrast, linear regression models, which assume that the data follow a Gaussian distribution, determine the standard error of the coefficient estimates and output confidence intervals. Since they allow us to understand the probabilistic nature of the data generation process, they are suitable method for inference. Also, a decision tree is one of the most widely used and practical methods for inductive inference. Particularly, Bayesian networks are popular for causal inference, since these models can be arranged to incorporate many assumptions about the data generation process.

Although there is no unified framework for ML interpretability, in general, the interpretability of the NIP ML methods can be improved by integrating them with the IP ML approaches. In addition to combining handcrafted features with latent variables, employing decision trees to encode all potential decision modes of the CNN in a coarse-to-fine manner as previously stated in Interpretability Improvement for Deep Learning, nomograms were employed to visualize and interpret SVM results^44,71^ and to combine a DL-based radiomics signature with clinical factors to improve the accuracy and interpretability of overall survival prediction.^48^ Moreover, other IP ML methods have also been used to improve the explanation of DL methods. For example, the problem of neural network structural learning was cast as a problem of Bayesian network structure learning, where a generative graph was learned, and its stochastic inverse was constructed resulting in a discriminative graph to simplify the neural network structure. Also it was proven that conditional-dependency relations among the latent variables in a generative graph are preserved in the class-conditional discriminative graph.^72^ In order to handle the exhaustive and empirical neural network parameterization process, a new deep Bayesian network architecture was proposed by adopting the principle of multilayer Bayesian network in order to make use of the edges’ significance, the causality, and the uncertainty of the Bayesian network for improving the meaningfulness of the hidden layers and the latent variable’s connections.^73^

As more patient-specific information is becoming available due to advances in imaging and biotechnology, the classical *p*(variables)>>*n*(samples) inference problem of statistical learning will become more challenging in the areas of personalized and adaptive radiation oncology. Therefore, more advanced data analytics will be deployed and the demand to integrate accuracy and interpretability will rise to cope with clinical practice needs in the field.^74^ Although different techniques are associated with distinct inherent limitations for radiation outcomes prediction, which include the independence assumption for features in logistic regression, the robustness in decision trees, the need for feature discretization in Bayesian networks, or the network configuration dependency in DL, our review shows that combining predictions among a handful of good, but different, IP and NIP models may result in better ML approaches to achieve higher accuracy and interpretability for radiation outcomes prediction.
